# Society Meetings

**Published:** 1909-09

**Authors:** 


### SOCIETY MEETINGS.

---

The American Association of Obstetricians and Gynecologists will hold its twenty-second annual meeting at the Hotel Anthony, Fort Wayne, Ind., Tuesday, Wednesday and Thursday, September 21, 22 and 23, 1909, under the presidency of Dr. William H. Humiston, of Cleveland, O. Dr. Miles F. Porter, 207 West Wayne, Fort Wayne, is chairman of the committee of arrangements, who should be addressed concerning hotel accommodations as well as other details of a local nature.

At 7 o'clock p. m. Wednesday, the annual dinner will be served at the Hotel Anthony. The cost of each cover will be $3.00, which will provide an excellent service, exclusive of wines and cigars.

Every member who contemplates attending the dinner is requested to notify the secretary and likewise designate the number of covers he wishes reserved. Fellows are entitled to invite guests who are not members.

### SCIENTIFIC PROGRAM.

The following is a list of papers offered up to August 25, 1909:

1. The president's address. William H. Humiston, Cleveland.
2. The Embryo abdominal surgeon with inadequate preparation and knowledge, J. H. Carstens, Detroit.
3. Some phases and case reports of puerperal sepsis, Hugo O. Pantzer, Indianapolis.
4. Drainage, J. F. Baldwin, Columbus.
5. Vaginal and abdominal cesarean section compared and contrasted, Miles F. Porter, Fort Wayne.
6. Title to be announced later. John F. Erdmann, New York.
7. When shall we operate for ruptured ectopic gestation? R. R. Huggins, Pittsburg.
8. The influence of pregnancy upon uterine myomata and the influence of myomata upon the progress of gestation and labor, E. Gustav Zinke, Cincinnati.
9. Nephrocoloptosis, with lantern demonstration, H. W. Longyear, Detroit.
10. A study of four hundred and thirty-six operations on the appendix with remarks. Edward J. Ill, Newark.
11. The new point in diagnosis between appendicitis and tubal diseases, Robert T. Morris, New York.
12. Terminal events in gallstone disease, Charles N. Smith. Toledo.
13. Operations upon handicapped patients, George W. Crile, Cleveland.
14. Some advance steps in the surgery of the colon, Charles A. L. Reed, Cincinnati.
15. A new technic for extirpation of the rectum with subsequent sphincteric control, Charles A. L. Reed, Cincinnati.
16. Some observations on the treatment of uterine cancer, L. S. McMurtry, Louisville.

17. Some personal experiences in gall-bladder surgery, Herman E. Hayd, Buffalo.

18. Is the routine exhibition of the pre-operative purge defensible? Edwin Walker, Evansville.

19. Specimen of calcareous degeneration of fibroid uterus, Walter B. Dorsett, St. Louis.

20. Title to be announced later. Ernst Jonas, St. Louis.

21. Gangrene of gall-bladder, M. A. Tate, Cincinnati.

22. Caries of the hyoid bone, J. E. Cannaday, Charleston.

23. An operation for cystocele that has given satisfactory results, Francis Reder.

24. Rupture of the uterus during labor. (A study based upon 78 cases from the three clinics of the New York Lying-in Hospital). Ralph Waldo Lobenstine (by invitation) New York.

25. Partum hemorrhage, with two quick methods of meeting the emergency, D. H. Stewart, New York.

26. (*a*) Presentation of specimens with brief reports; (*b*) Why we have to drain in pelvic and abdominal surgery; (*c*) Who is responsible for the abdominal junk demanding reoperations? Joseph Price, Philadelphia.

27. How can we best educate women to seek early relief for carcinoma of the uterus? Carlton C. Frederick, Buffalo.

28. Observations on blood transfusion, John D. S. Davis, Birmingham.

29. Malignant tumor of undescended testicle, Orange G. Pfaff, Indianapolis.

30. Title to be announced. John Young Brown, St. Louis.

31. Surgical treatment of tumors of the bladder, John W. Keefe, Providence.

32. Title to be announced. N. Stone Scott, Cleveland.

33. Phlegmasia dolens in connection with ovarian tumor, William A. B. Sellman, Baltimore.

34. Chylous cyst of the mesentery. Charles E. Congdon, Buffalo.

All members of the medical profession and medical students are cordially invited to attend the scientific sessions. William H. Humiston, president, Cleveland; William Warren Potter, secretary, Buffalo.

---

THE Lake Keuka Medical and Surgical Association held its tenth annual meeting at Grove Springs, Lake Keuka, N. Y., Thursday and Friday, July 15 and 16, 1909, under the direction of the following-named officers: president, O. J. Hallenbeck, Canandaigua; vice-president, Ross G. Loop, Elmira; secretary and treasurer,

H. B. Nichols, Pulteney; committee of arrangements, P. L. Alden, Hammondsport; E. M. Sherer, Penn Yan; R. G. Lawrence, Hammondsport; W. W. Bachman, Prattsburg; registration committee, W. W. Smith, Avoca. The program contained the names of Irving P. Lyon and William C. Krauss, of Buffalo.

---

The Psychological Congress that met in Geneva, Switzerland, the first week in August, 1909, accepted by acclamation the American invitation to hold its next congress, in 1913, in Boston. The following officers were chosen: Honorary president, William James, of Cambridge, Mass.; president, James Mark Baldwin, of Baltimore, and vice-presidents, Edward Bradford Titchener Sage, professor of psychology at Cornell University, and James McKeen Cattell, professor of psychology at Columbia University.

